# Hepatocyte toll-like receptor 4 mediates lipopolysaccharide-induced hepcidin expression

**DOI:** 10.1038/emm.2017.207

**Published:** 2017-12-08

**Authors:** Yong-Soo Lee, Yong-Hoon Kim, Yoon Seok Jung, Ki-Sun Kim, Don-Kyu Kim, Soon-Young Na, Ji-Min Lee, Chul-Ho Lee, Hueng-Sik Choi

**Affiliations:** 1 National Creative Research Initiatives Center for Nuclear Receptor Signals and Hormone Research Center, School of Biological Sciences and Technology, Chonnam National University, Gwangju, Republic of Korea; 2 Laboratory Animal Resource Center, Korea Research Institute of Bioscience and Biotechnology, Daejeon, Republic of Korea; 3 Department of Functional Genomics, University of Science and Technology (UST), Daejeon, Republic of Korea; 4 Department of Molecular Biotechnology, Chonnam National University, Gwangju, Republic of Korea

## Abstract

Hepcidin expression is induced by inflammatory molecules such as lipopolysaccharide (LPS) via a macrophage-mediated pathway. Although hepatocytes directly respond to LPS, the molecular mechanism underlying toll-like receptor (TLR)-dependent hepcidin expression by hepatocytes is mostly unknown. Here we show that LPS can directly induce the mRNA expression and secretion of hepcidin by hepatocytes via TLR4 activation. Using hepatocytes deficient in TLR4, myeloid differentiation factor 88 (MyD88) and TIR domain-containing adaptor inducing interferon-β (TRIF), we demonstrated that LPS-induced hepcidin expression by hepatocytes is regulated by its specific receptor, TLR4, via a MyD88-dependent signaling pathway. Hepcidin promoter activity was significantly increased by MyD88-dependent downstream signaling molecules (interleukin-1 receptor-associated kinase (IRAK) and tumor necrosis factor receptor-associated factor 6 (TRAF6), which activate c-Jun N-terminal kinase (JNK) and activator protein-1 (AP-1). We then confirmed that LPS stimulation induced the phosphorylation of JNK and c-Jun, and observed strong occupancy of the hepcidin promoter by c-Jun. Promoter mutation analysis also identified the AP-1-binding site on the hepcidin promoter. Finally, bone marrow transplantation between wild-type and TLR4 knockout mice revealed that hepatic TLR4-dependent hepcidin expression was comparable to macrophage TLR4-dependent hepcidin expression induced by LPS. Taken together, these results suggest that TLR4 expressed by hepatocytes regulates hepcidin expression via the IRAK–TRAF6–JNK–AP-1 axis.

## Introduction

During infection, microbes acquire iron from the host; however, the host resists infection by sequestering iron.^1^ The iron-regulatory hormone hepcidin is an important orchestrator of this host response. Hepcidin, initially identified as an antimicrobial peptide, has an important role in regulating iron homeostasis.^2, 3^ It is synthesized in the liver and regulates the trans-membrane transporter of iron, ferroportin, in enterocytes, macrophages and hepatocytes. Once hepcidin binds to ferroportin, the complex is internalized and degraded, thereby inhibiting iron export from cellular stores.^4^ Hepcidin is regulated by a variety of stimuli, including iron status, inflammation and hypoxia.^5, 6^ The transcription of the hepcidin gene in response to iron status is regulated by the hemojuvelin/bone morphogenetic protein 6/SMAD (HJV/BMP6/SMAD) signaling pathway;^7, 8^ in addition, hepcidin induction by inflammatory stimuli is dependent on the stimulation of the JAK2/STAT3 signaling pathway.^9^ Studies show that the Hippel–Lindau/hypoxia-inducible transcription factor pathway functions as an essential link between iron homeostasis and hepcidin regulation in the mechanism underlying hypoxia-induced downregulation of hepcidin expression.^10^ As mentioned above, hepcidin was first identified as a liver-expressed antimicrobial peptide that has antimicrobial and antifungal properties.^2, 3, 11^ However, it has recently been reported that hepcidin has an important role in the innate immune responses of many living organisms by controlling iron levels, rather than through direct bactericidal effects.

The innate immune system is the first line of defense against infection and is involved in various inflammatory processes. Toll-like receptors (TLRs) are pattern recognition receptors, and signaling through TLRs leads to the expression of both pro-inflammatory and anti-inflammatory cytokines.^12, 13^ To date, 13 TLRs have been identified, although TLR12 and TLR13 are not present in the human genome.^14^ Among the TLRs, TLR4 is the key regulator of bacterial clearance and the host inflammatory response. TLR4 is activated by its ligand and by three different extracellular proteins: lipopolysaccharide (LPS)-binding protein; CD14; and myeloid differentiation protein 2. These molecules are expressed on cell membranes.^15^ After activation, the TLR4 complex undergoes oligomerization in the cytosol and recruits the downstream adaptor proteins myeloid differentiation factor 88 (MyD88), MyD88-adaptor-like/TIR domain-containing adaptor protein,^16^ TIR domain-containing adaptor inducing interferon-β (TRIF) and TRIF-related adaptor molecule.^17, 18^ There are two different TLR4 signaling pathways that differ based on the intracellular adaptor molecules recruited. The MyD88-dependent signaling pathway is mediated by interleukin (IL)-1 receptor-associated kinase (IRAK), tumor necrosis factor receptor-associated factor 6 (TRAF6) and transforming growth factor-β-activated kinase 1, which activate the downstream IκB kinase or mitogen-activated protein kinase pathways.^19^ These events lead to the activation of the transcription factors nuclear factor-κB (NF-κB) or activator protein-1 (AP-1), which regulate the expression of inflammation-related genes. In addition, phosphatidylinositol 3-kinase and AKT are important factors downstream of MyD88; these molecules also regulate NF-κB activation.^20^ Meanwhile, the MyD88-independent signaling pathway is mediated by TRIF, which activates interferon (IFN) regulatory factor 3 and induces expression of IFNβ and IFN-responsive genes.^21^

Because the host immune response restricts iron availability to invading pathogens, hepcidin is induced by inflammatory cytokines that are produced by macrophages expressing TLRs. However, several studies have shown that hepatocytes also express TLRs.^22, 23, 24^ Here we hypothesized that TLRs expressed by hepatocytes have a direct role in regulating hepcidin gene expression because the liver is constantly exposed to endotoxins produced by gut bacteria, and hepatocytes constitute the majority of liver cells. To demonstrate this idea, we examined LPS-induced hepcidin expression in the AML12 cell line and in primary hepatocytes from various TLR4 signal-related gene-deficient mice. The results showed that LPS-induced hepatic TLR4-dependent hepcidin expression is of functional significance.

## Materials and methods

### DNA constructs

The reporter plasmid containing the mouse hepcidin promoter (−982/+84) was described previously.^25^ For overexpression, the following DNA constructs were used: pCMV-SPORT6-Myd88; pEF1alpha-IRAK1; pEAK12-TRAF6; pcDNA3-JNK1; pcDNA3-JNK2; pcDNA3–c-jun; pcDNA3-c-fos; and pCMV-SPORT6-p65. The AP-1 mutant (mut, −89 TGAGTCA −83 to AAAGTCA) on the mouse hepcidin promoter-Luc reporter was generated using the QuikChange II site-directed mutagenesis kit (Stratagene, La Jolla, CA, USA).

### Reagents

LPS (*Escherichia coli* 026:B6, L2654, Sigma Aldrich, St Louis, MO, USA), BMP6 (6325-BM, R&D Systems, Minneapolis, MN, USA) and IL-6 (CYT-213, PROSPEC, Ness-Ziona, Israel) were dissolved in manufacturer-recommended solvents. The TLR4 signaling inhibitors polymyxin B and CLI095 were purchased from InvivoGen (San Diego, CA, USA) and dissolved in the recommended solvents. Specific inhibitors of MEK (PD98059), phosphatidylinositol 3-kinase (LY294002), JNK (SP600125), p38 mitogen-activated protein kinase (SB203580) and NF-κB (Bay11-7082) were purchased from Cell Signaling Technology (Danvers, MA, USA) and dissolved in the recommended solvents.

### Cell culture and transient transfection

293T (human embryonic kidney cells) cells were maintained in Dulbecco’s modified Eagle’s medium supplemented with 10% fetal bovine serum and antibiotics. AML12 (immortalized mouse hepatocyte) cells were cultured in Dulbecco’s modified Eagle’s medium/F-12 medium supplemented with 10% fetal bovine serum, an insulin–transferrin–selenium mixture, dexamethasone (40 ng ml^−1^) and antibiotics. All cell lines were maintained in a humidified atmosphere containing 5% CO_2_ at 37 °C. Transient transfections were carried out using Lipofectamine 2000 (Invitrogen, Carlsbad, CA, USA), according to the manufacturer’s instructions. Cells were co-transfected with a reporter plasmid (0.2 μg per well) and the indicated expression vector (0.4 μg per well). A pCMV-β-galactosidase plasmid (0.1 μg) was co-transfected as an internal control. The total amount of DNA was adjusted to 0.8 μg per well by addition of each corresponding empty vector. Cells were collected 24 h after transfection. Luciferase activity was normalized to β-galactosidase activity. Data are representative of at least three independent experiments.

### Isolation and culture of primary mouse hepatocytes

Mouse primary hepatocytes were isolated from the livers of 8-week-old male C57BL/6 mice and 8-week-old male *Tlr4*, *Myd88* or *Trif* knockout (KO) mice (Jackson Laboratory, Bar Harbor, ME, USA). Briefly, mice were anesthetized with Zoletile (Virbac, Carros, France), and the liver was exposed surgically, perfused with resuspension buffer and then perfused with collagenase solution. Subsequently, the liver was finely chopped in a Petri dish and then passed through an 85 μm pore mesh filter. Hepatocytes were collected by centrifugation at 800 × *g* for 2–5 min at 4 °C. Hepatocyte viability was assessed in a trypan blue exclusion assay: viability was consistently >85%. Hepatocytes were then seeded onto 60 mm dishes coated with collagen.

### Quantitative real-time PCR analysis

Total RNA was isolated from AML12 cells, mouse primary hepatocytes or mouse livers using TRIzol reagent (Invitrogen), according to the manufacturer’s instructions, and cDNAs generated using the Maxime RT PreMix Kit (iNtRON Biotechnology, Seongnam-si, Korea) were analyzed using the Applied Biosystems StepOnePlus real-time PCR system (Applied Biosystems, Waltham, MA, USA) with Power SYBR Green PCR Master Mix (Applied Biosystems). All data were normalized to actin expression. The following primers were used: Hamp1 (mouse hepcidin), forward 5′-TGCCTGTCTCCTGCTTCTCCT-3′ and reverse 5′-GATGGGGAAGTTGGTGTCTC-3′ inducible nitric oxide synthase, forward 5′-GGGCAGCCTGTGAGACCTT-3′ and reverse 5′-CATTGGAAGTGAAGCGTTTCG-3′ and β-actin, forward 5′-TCTGGCACCACACCTTCTAC-3′ and reverse 5′-TCGTAGATGGGCACAGTGTGG-3′.

### Western blot analysis

Whole-cell extracts of cell lines or mouse tissues (spleen) were prepared using RIPA buffer (Elpis-Biotech, Daejeon, Korea). Proteins were separated on 10% SDS-polyacrylamide gel electrophoresis gels and transferred to nitrocellulose membranes. The membranes were then probed with the indicated antibodies (JNK, phospho-JNK, c-Jun, phospho-c-Jun and tubulin antibodies: Cell Signaling Technology; an'd ferroportin antibody (SLC40A1): Invitrogen). Immunoreactive proteins were visualized using an Amersham ECL kit (GE Healthcare, Piscataway, NJ, USA), according to the manufacturer’s instructions.

### Measurement of hepcidin and iron levels

Hepcidin was measured using a mouse hepcidin (Hepc) enzyme-linked immunosorbent assay (ELISA) kit (CUSABIO, catalog; CSB-E14395m, Wuhan, Hubei Province, China), according to the manufacturer’s instructions. Iron was measured using an iron assay kit (Abcam, ab83366, Cambridge, MA, USA), according to the manufacturer’s instructions.

### Chromatin immunoprecipitation assay

The chromatin immunoprecipitation assay was performed according to the manufacturer’s protocol (Upstate Biotechnology, Lake Placid, NY, USA). Briefly, AML12 cells were treated with 1 μg of LPS for 24 h, fixed with 1% formaldehyde and then collected. Soluble chromatin was immunoprecipitated with an anti-c-Jun antibody (#9165, Cell Signaling Technology). After recovering DNA, PCR was performed using primers encompassing the AP-1-binding region on the mouse hepcidin promoter (forward 5′-CTGGCTGTAGGTGACACAAC-3′ and reverse 5′-AAGGACTTGTGTGGTGGCTG-3′). The size of the amplified PCR product was 193 bp.

### Animals and bone marrow transplantation studies

Male 8-week-old *Tlr4*, *Myd88* or *Trif* KO mice on a C57BL/6 background and wild-type (WT) C57BL/6 control mice were obtained from the Jackson Laboratory and kept in a specific pathogen-free facility. Before experiments, mice were acclimatized to a 12 h light/dark cycle at 22±2 °C for 2 weeks and allowed unlimited access to food and water. Bone marrow chimeric mice were generated by injecting 3 × 10^6^ bone marrow cells into sublethally irradiated recipients (900 rad), followed by intravenous injection of LPS (500 μg kg^−1^) 8 weeks later. All mice were killed by CO_2_ asphyxiation. All animal experiments were approved by the Institutional Animal Use and Care Committee of the Korea Research Institute of Bioscience and Biotechnology and were performed in accordance with the Guide for the Care and Use of Laboratory Animals published by the US National Institutes of Health.

### Statistical analysis

Data are expressed as the mean±s.e. Statistical analysis was performed using two-tailed Student’s *t*-test. Differences were considered significant at *P*<0.05.

## Results

### LPS induces hepcidin expression in hepatocytes

**Figure 1:**
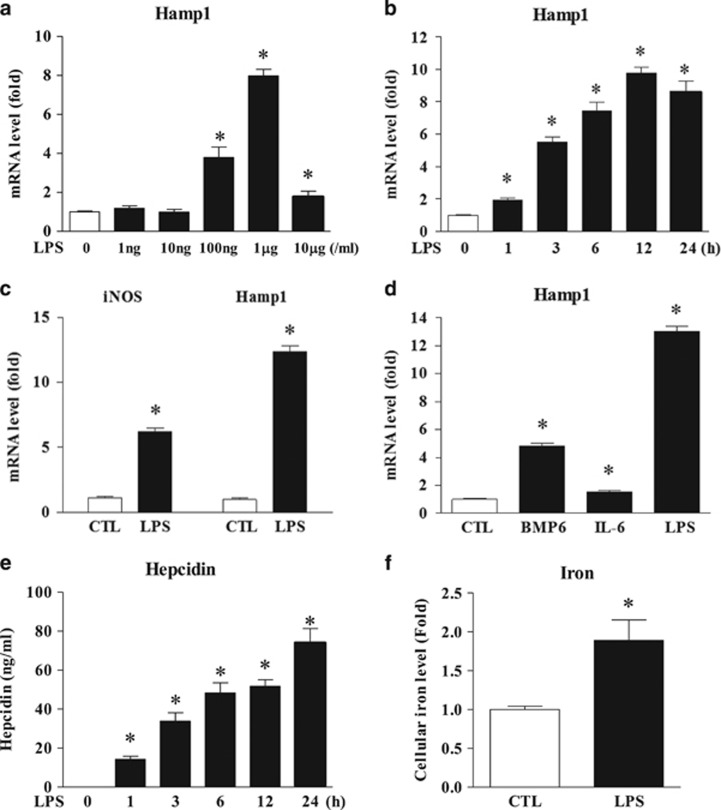
LPS induces hepcidin expression by hepatocytes. (**a**) AML12 cells were treated with different concentrations of LPS for 24 h, and the expression of mouse hepcidin (Hamp1) mRNA was measured by quantitative real time polymerase chain reaction (qRT-PCR). (**b**) AML12 cells were treated with LPS (1 μg ml^−1^) for the designated times. (**c**) Expression of mRNA encoding inducible nitric oxide synthase (iNOS) and Hamp1 in AML12 cells treated with 1 μg ml^−1^ LPS for 24 h. (**d**) AML12 cells were treated for 12 h with BMP6 (20 ng ml^−1^), IL-6 (20 ng ml^−1^) or LPS (1 μg ml^−1^), and the expression of Hamp1 mRNA was measured by qRT-PCR. (**e**) Hepcidin concentration in cell culture medium from LPS (1 μg ml^−1^)-treated AML12 cells. (**f**) Iron concentration in AML12 cells treated with LPS (1 μg ml^−1^) for 24 h.

To assess the effect of LPS on hepcidin expression in hepatocytes, we first selected the optimal concentration of LPS to induce hepcidin expression. As shown in Figures 1a and b, 1 μg ml^−1^ of LPS elicited the highest hepcidin mRNA expression in AML12 cells, and the LPS-mediated hepcidin expression increased in a time-dependent manner for 24 h. To confirm the LPS responsiveness of hepatocytes, we then examined the expression of inducible nitric oxide synthase (iNOS), which is induced by LPS challenge.^26, 27^ As shown in Figure 1c, levels of iNOS mRNA, as well as those of hepcidin, were markedly elevated upon exposure to LPS, suggesting that LPS acts directly on hepatocytes to invoke target gene expression. We also confirmed that compared with BMP6 and IL-6, which were used as positive controls, LPS effectively induced hepcidin mRNA expression (Figure 1d). To verify the substantial hepcidin secretion, we performed an ELISA using culture medium from LPS-treated AML12 cells. Hepcidin secretion by LPS-treated hepatocytes was significantly higher than that by control cells (without LPS; Figure 1e). Moreover, LPS-induced hepcidin expression led to a marked increase in the cellular iron concentration (Figure 1f). Taken together, these results suggest that LPS induces hepcidin expression and secretion by hepatocytes.

### LPS induces expression of hepcidin by hepatocytes through the TLR4–MyD88-dependent signaling pathway

**Figure 2:**
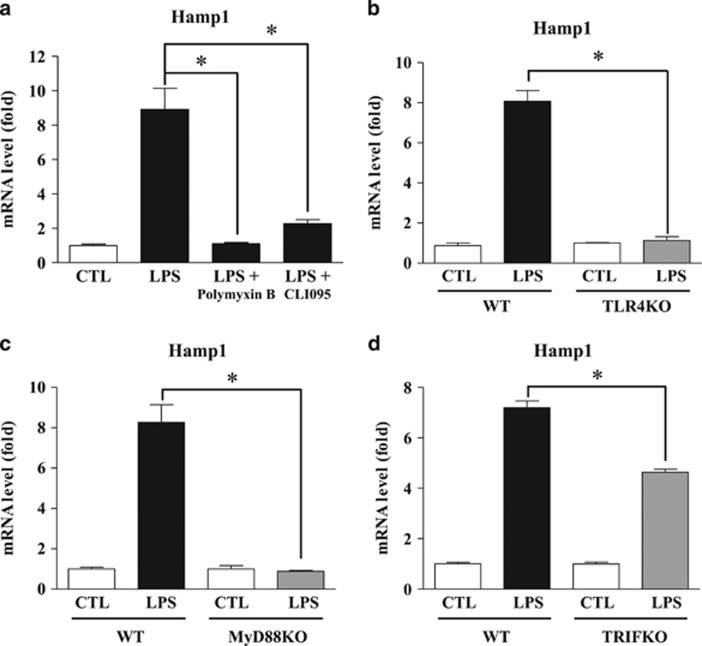
LPS induces expression of hepcidin by hepatocytes via the TLR4 pathway. (**a**) Expression of Hamp1 mRNA in AML12 cells treated with LPS (1 μg ml^−1^) in the presence or absence of the TLR4 inhibitors polymyxin B (100 μg ml^−1^) or CLI095 (3 μM) for 24 h. Each inhibitor was used at the manufacturer’s recommended working concentration. (**b**–**d**) Expression of Hamp1 mRNA in WT versus TLR4 KO mice (**b**), MyD88 KO mice (**c**) and TRIF KO mice (**d**). Isolated mouse primary hepatocytes were treated with LPS (1 μg ml^−1^) for 24 h.

LPS is a major pathogen-activated molecular pattern expressed on the outer membrane of Gram-negative bacteria; thus, it is specifically recognized by TLR4 and elicits an immune response.^12^ To further confirm whether LPS-induced hepcidin expression in hepatocytes depends on the specific LPS receptor, we exposed cells to the following specific inhibitors of TLR4 signaling: polymyxin B, a cyclic cationic polypeptide antibiotic that inhibits the binding of LPS to TLR4; and CLI095, a TLR4 intracellular signaling inhibitor. As shown in Figure 2a, LPS-induced hepcidin expression was significantly reduced by both polymyxin B and CLI095 in AML12 cells. Interestingly, LPS did not induce hepcidin expression in primary hepatocytes isolated from TLR4 KO mice (Figure 2b). The TLR4 signaling pathway actually comprises two signaling pathways, the MyD88-dependent and the MyD88-independent pathways; the latter is mediated by TRIF.^16^ To ascertain which signaling pathway is involved in LPS-induced hepcidin expression by hepatocytes, we compared hepcidin expression in primary hepatocytes isolated from MyD88 KO and TRIF KO mice after challenge with LPS. As shown in Figure 2c, hepatocytes isolated from MyD88 KO mice did not respond to LPS stimulation, as well as those isolated from TLR4 KO mice. However, LPS-induced hepcidin expression in hepatocytes isolated from TRIF KO mice was significantly augmented, although the induced hepcidin level was less than that observed in cells from WT mice (Figure 2d). These results indicate that LPS-induced expression of hepcidin by hepatocytes is regulated by the TLR4–MyD88-dependent signaling pathway.

### JNK has a key role in TLR4–MyD88-mediated downstream signaling for hepcidin gene expression in hepatocytes

**Figure 3:**
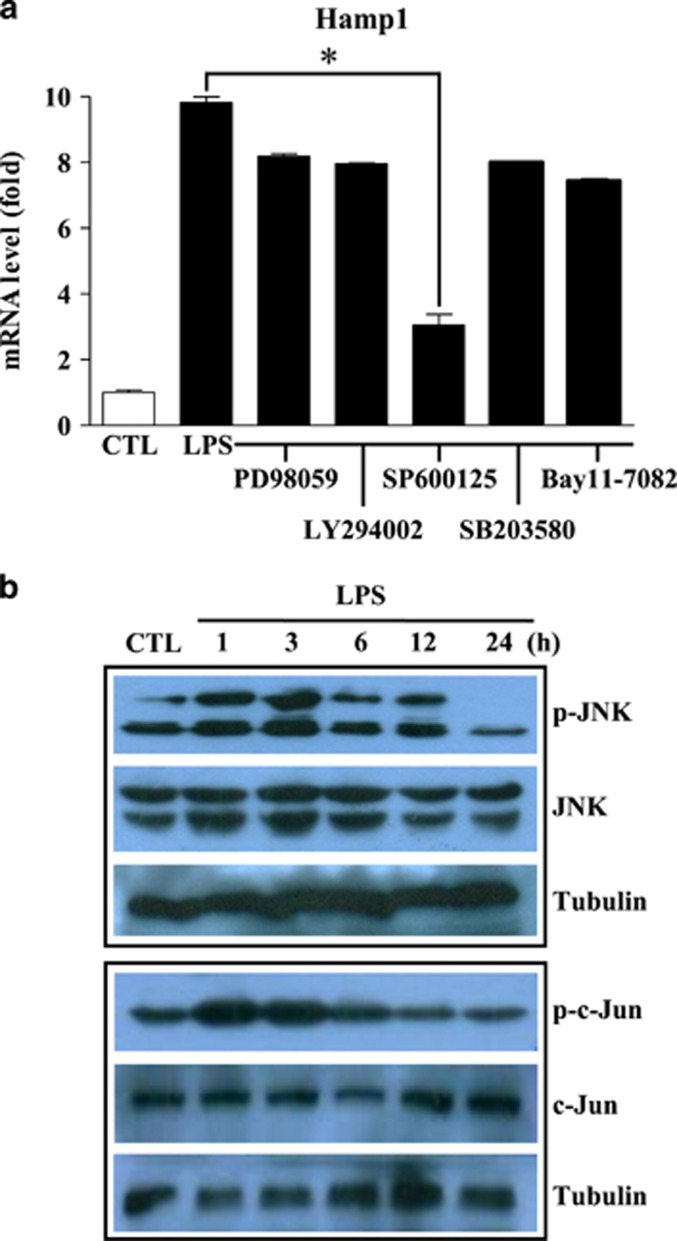
LPS induces the expression of hepcidin in hepatocytes by activating JNK and activator protein-1 (AP-1). (**a**) Expression of Hamp1 mRNA in AML12 cells treated with PD98059 (a MEK inhibitor), LY294002 (a PI3K inhibitor), SP600125 (a JNK inhibitor), SB203580 (a p38 MAP kinase inhibitor) or Bay11-7082 (a NF-κB) inhibitor), all at 5 μM. Cells were exposed to each inhibitor for 1 h before treatment with LPS (1 μg ml^−1^) for 4 h. (**b**) LPS activates JNK and AP-1 in hepatocytes. AML12 cells were treated with LPS (1 μg ml^−1^) for the designated times. Cell lysates were prepared and examined by western blotting with anti-phospho-JNK, anti-phospho-c-jun, anti-JNK and anti-c-jun antibodies. An anti-alpha tubulin antibody was used as an internal control. The blots were prepared in duplicate for each independent western blot for JNK and c-Jun.

MyD88 activates the downstream signaling molecule IRAK, which then interacts with TRAF6.^19^ The IRAK–TRAF6 complex activates IκB kinase or mitogen-activated protein (MAP) kinase, which in turn leads to the activation of NF-κB or AP-1, respectively. In addition, phosphatidylinositol 3-kinase (PI3K) and AKT are also important factors downstream of MyD88 that regulate NF-κB activation.^20^ Therefore, we next used various signal inhibitors (PD98059 (MEK), LY294002 (PI3K), SP600125 (JNK), SB203580 (p38 MAP kinase) and Bay11-7082 (NF-κB) to identify the signaling molecule(s) directly involved in LPS-induced hepcidin expression by hepatocytes. As shown in Figure 3a, LPS-induced hepcidin expression by AML12 cells was significantly repressed by the JNK inhibitor, SP600125, suggesting that JNK is the major target molecule of MyD88 signaling via LPS-TLR4 in hepatocytes. Phosphorylation and activation of JNK leads to the phosphorylation of serine residues within the N-terminal portion of c-Jun, which in turn activates target genes harboring AP-1 enhancer sequences in their promoter regions.^28^ Western blot analysis confirmed phosphorylation and activation of JNK and its target molecule, c-jun, after LPS treatment of hepatocytes (Figure 3b). Taken together, these results demonstrate that JNK and AP-1 are directly targeted by the LPS–TLR4–MyD88 signaling pathway in hepatocytes.

### JNK-dependent activation of AP-1 directly regulates hepcidin promoter activity in hepatocytes

**Figure 4:**
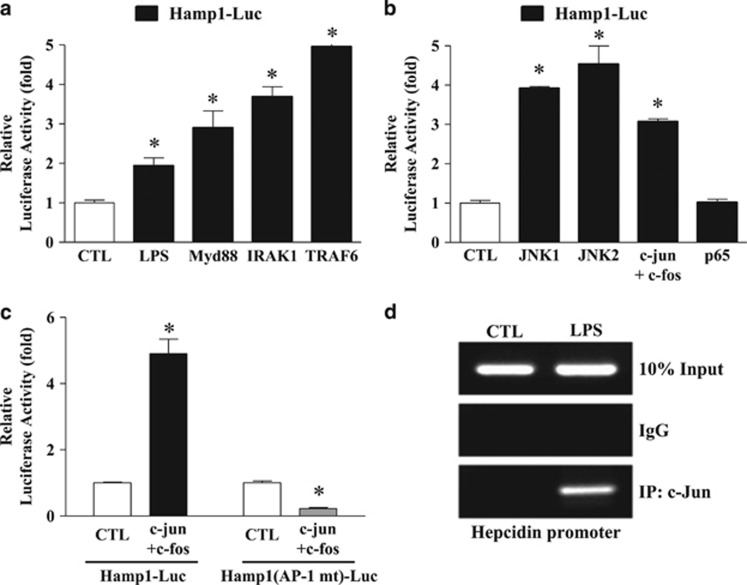
The TLR4 signaling pathway regulates the hepcidin promoter. (**a**) Effect of TLR4 downstream signaling molecules on hepcidin promoter activity. AML12 cells were co-transfected with a hepcidin promoter luciferase reporter (Hamp1-Luc; 200 ng) and each of the indicated signaling molecules (MyD88, IRAK1 and TRAF6; 400 ng). LPS (1 μg ml^−1^) treatment for 24 h before cell harvest was used as a positive control. (**b**) Effect of JNK–AP-1 signaling on hepcidin promoter activity. AML12 cells were co-transfected with Hamp1-Luc (200 ng) and JNK (1 or 2) or AP-1 (c-jun+c-fos) or NF-κB; p65; all at 400 ng). (**c**) Regulation of hepcidin promoter activity by activator protein-1 (AP-1). 293T cells were co-transfected with the wild-type hepcidin promoter, Hamp1-Luc (200 ng), or with an AP-1-binding site-specific mutated promoter, Hamp1 (AP-1 mt)-Luc (200 ng) and AP-1 (c-jun+c-fos; 400 ng). (**d**) Binding of AP-1 to the hepcidin promoter. Chromatin immunoprecipitation assay using an anti-c-jun antibody and the primers harboring the AP-1-binding site in LPS-treated (for 24 h) AML12 cells.

To further examine whether the molecules downstream of MyD88 regulate hepcidin expression by hepatocytes, we transiently transfected AML12 cells and assessed the hepcidin promoter activity induced by overexpression of IRAK1 and TRAF6. As shown in Figure 4a, IRAK1 and TRAF6 increased hepcidin promoter-driven luciferase activity as efficiently as MyD88. We also confirmed that JNK and AP-1 (c-jun/c-fos) led to a significant increase in hepcidin promoter activity, whereas the NF-κB component p65 did not (Figure 4b). We then examined whether AP-1 is a major signaling molecule involved in activating the hepcidin promoter. As shown in Figure 4c, AP-1 caused a significant increase in the activity of the WT hepcidin promoter but did not activate the AP-1-binding site-mutated hepcidin promoter. Furthermore, a chromatin immunoprecipitation assay showed that LPS stimulation led to strong occupancy of the hepcidin promoter by c-Jun (Figure 4d). Taken together, these results indicate that the JNK–AP-1 axis directly regulates the hepcidin promoter.

### Hepatic TLR4 has an important role in LPS-induced hepcidin expression

**Figure 5:**
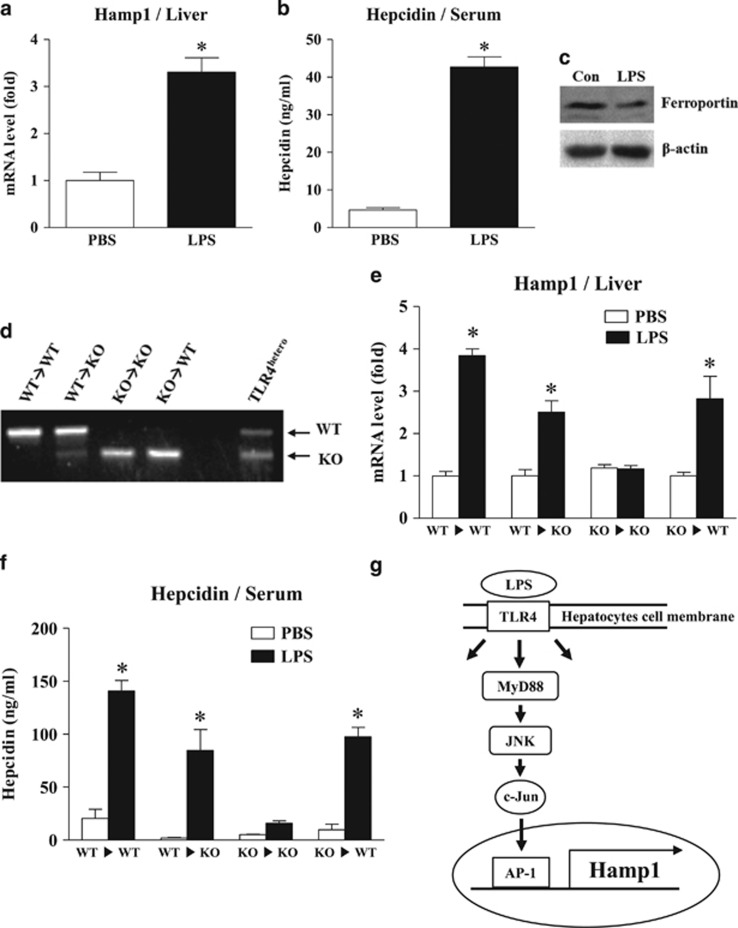
Role of TLR4 in LPS-induced hepcidin expression by hepatocytes. (**a**–**c**) LPS-mediated hepcidin expression in the livers of mice. Eight-week-old male C57BL/6 mice (*n*=4) received an intravenous injection of LPS (500 μg kg^−1^). Six hours later, expression of Hamp1 mRNA from livers was measured by qRT-PCR (**a**), and the hepcidin concentration in the serum was measured by ELISA (**b**). The effect of LPS-induced hepcidin on ferroportin degradation in the spleen was verified by western blot analysis using an anti-ferroportin antibody. All the samples (*n*=4) were combined for western blot analyses (**c**). (**d**) Genotyping of genomic DNA isolated from the peripheral blood of bone marrow-transplanted chimeric mice. LPS-induced expression of hepcidin mRNA in mice receiving a bone marrow transplant (*n*=3–4). (**e**) Eight weeks after bone marrow transplantation, chimeric mice were challenged with LPS for 6 h, and expression of hepcidin mRNA in the liver was measured. (**f**) Concentration of LPS-induced hepcidin in serum from chimeric mice as measured by ELISA. (**g**) Proposed signaling pathway for LPS-induced hepcidin expression in hepatocytes.

To evaluate the role of hepatic TLR4 in LPS-induced hepcidin expression, we first confirmed that LPS challenge for 6 h was sufficient to induce hepcidin expression in the mouse liver and observed that this treatment led to a marked increase in serum hepcidin level (Figures 5a and b). Importantly, LPS-induced hepcidin caused ferroportin degradation in the spleen, which is an organ well known for hepcidin-dependent iron regulation (Figure 5c). We next generated TLR4-chimeric mice using irradiation and bone marrow transplantation, which replaced the resident macrophages. We then confirmed that the chimeric mice had been generated appropriately (Figure 5d). Eight weeks after bone marrow transplantation, the chimeric mice were stimulated with LPS for 6 h, and the livers were isolated to measure the levels of hepcidin mRNA. As shown in Figure 5e, compared with control mice (WT ▸ WT), TLR4 KO bone marrow-transplanted WT mice (KO ▸ WT) exhibited comparable levels of hepcidin mRNA expression. ELISA also confirmed that the levels of hepcidin secreted by cells from these chimeric mice were comparable to those secreted by cells from control mice (Figure 5f). These results imply that macrophages are not the only cells that can respond to LPS to induce hepcidin expression and secretion by hepatocytes. TLR4 WT bone marrow-transplanted TLR4 KO mice (WT ▸ KO) responded to LPS and produced hepcidin, suggesting that the transplanted macrophages responded to LPS and activated hepatocytes to produce hepcidin. Taken together, these data indicate that TLR4 expressed by hepatocytes also has a role in LPS-induced hepcidin production and that both macrophages and hepatocytes are major regulators of hepcidin levels.

## Discussion

The liver is a major organ and filters toxins produced by microorganisms in the gastrointestinal tract or by injured organs via the portal circulation. It also has an important role in producing the iron-regulatory protein hepcidin. In the liver, inflammatory responses are mainly mediated by resident macrophages, called Kupffer cells, which are the major cells involved in most biological responses to LPS through the TLR4 signaling pathway.^24^ Reports suggest that inflammation-mediated expression of hepcidin is regulated by pro-inflammatory cytokines secreted by Kupffer cells via TLR signaling.^9, 29, 30^ However, hepatocytes comprise the largest cell population in the liver; as such, they are constantly exposed to endotoxins derived from other organs. Moreover, hepatocytes also express TLR4 and respond to agonists such as LPS and saturated fatty acids,^23, 31^ suggesting that direct activation of TLR4 on hepatocytes by LPS induces the expression of hepcidin, which then regulates iron levels.

Here we examined how LPS affects hepatocyte function and hepcidin expression. The data indicate that hepatocytes respond to LPS and express hepcidin independently of macrophages. LPS-induced hepcidin expression by hepatocytes was induced rapidly and resulted in hepcidin levels comparable to those observed in macrophage-dependent hepcidin expression by hepatocytes. Moreover, direct exposure of hepatocytes to LPS was sufficient to induce hepcidin expression at levels comparable to those induced by BMP6 or IL-6, both well-known hepcidin inducers. These results demonstrate that LPS is a potent inducer of hepcidin in hepatocytes and that hepatic TLR4 is the primary signaling receptor responsible for hepcidin production. In particular, the bone marrow transplantation data demonstrated that macrophages are not the only cell type that is responsive to LPS in terms of hepcidin expression and secretion by hepatocytes. These results suggest that macrophage-independent hepcidin induction, in addition to macrophage-dependent hepcidin expression, has a role in systemic iron homeostasis. In the presence of LPS, macrophages produce pro-inflammatory cytokines such as IL-1β and IL-6, which then stimulate hepatocytes to produce hepcidin.^29^ However, when we consider the time required for a cell to produce hepcidin, a direct hepatocyte response to LPS would be a more effective and time-saving strategy for an organism. As a primary defense organ, the liver removes various toxins or microorganisms from the blood; therefore, hepatocytes, which are the most common cell type in the liver, may utilize this effective and time-saving strategy when exposed to high levels of LPS.

With respect to the molecular mechanism underlying hepcidin expression by LPS-exposed hepatocytes, it is clear that stimulation through the LPS–TLR4 axis depends on the MyD88-dependent signaling pathway, because primary hepatocytes from TLR4 KO and MyD88 KO mice did not induce hepcidin expression in response to LPS. In this context, the pathway is similar to that used by Kupffer cells in which activation of the MyD88-dependent signaling pathway leads to the expression of IL-6 and the subsequent stimulation of hepatocytes to produce hepcidin. However, the experiments with various signaling inhibitors revealed that JNK is the key regulator of hepcidin expression in LPS-stimulated hepatocytes. A previous study reported that serum stimulated hepcidin transcription via AP-1 binding to the hepcidin promoter.^32^ In our study, we found that the hepatic TLR4 signaling pathway activated JNK and induced the binding of AP-1 to the hepcidin promoter in the absence of macrophage-mediated cytokine stimulation. Another study also suggested that alcohol regulates hepcidin expression via TLR4–NF-κB signaling.^33^ Collectively, these data indicate that different stimuli utilize different signaling molecules to induce hepcidin, even though the stimulation signal is always transmitted via TLR4.

In summary, we found that LPS-exposed hepatocytes can regulate hepcidin expression via the LPS–TLR4–MyD88–JNK-AP-1 signaling pathway independently of liver macrophages (Figure 5g). TLR4 is the key regulator of bacterial clearance and the host inflammatory response; thus, considering that hepcidin is regulated by inflammation and that the liver is the central organ for systemic iron regulation, direct and acute responses of hepatocytes to pathogens are substantial and important events that trigger inflammation-mediated iron homeostasis. However, further studies are needed to identify the differences between macrophage-dependent and macrophage-independent mechanisms of hepcidin regulation.

## Publisher's note:

Springer Nature remains neutral with regard to jurisdictional claims in published maps and institutional affiliations.
